# Supplementary material for: Epidemiology and Genetic Analysis of SARS-CoV-2 in Myanmar during the Community Outbreaks in 2020
Source: Viruses. 2022 Jan 27;14(2):259. doi: 10.3390/v14020259 (PMC8875553; doi:10.3390/v14020259)

(A) Number of COVID-19 confirmed cases in Rakhine State

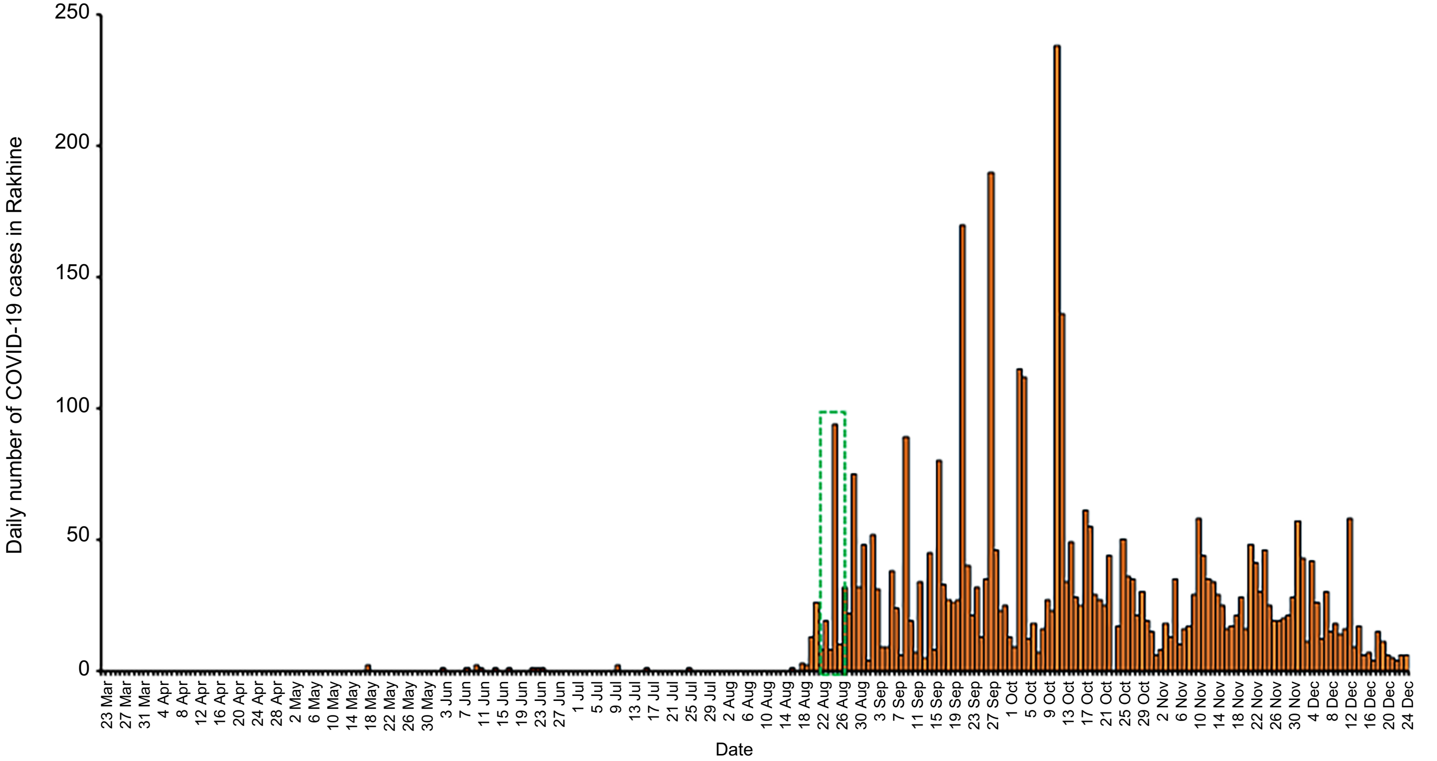

(B) Number of COVID-19 death cases in Rakhine State

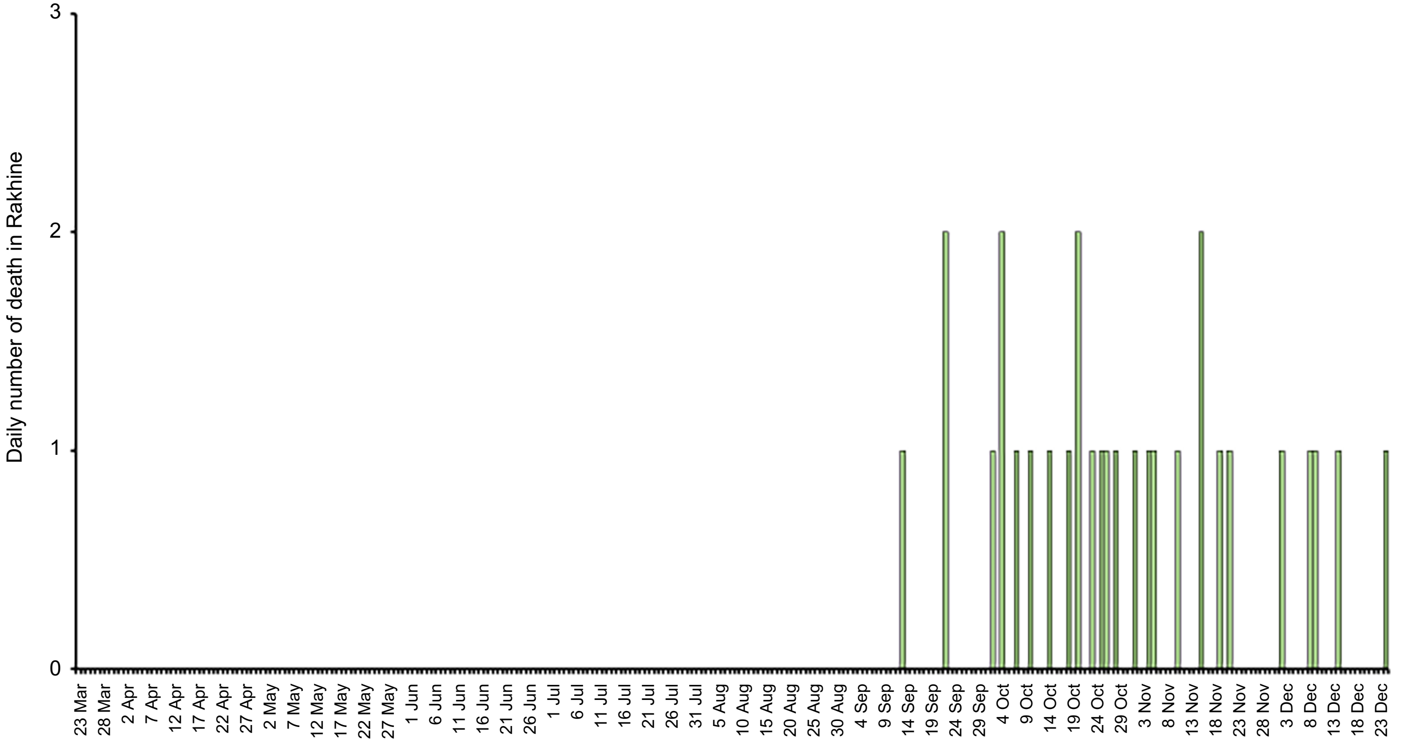

Supplement: Supplementary file 1 [file viruses-14-00259-s001.zip › FigureS2.pdf]
