# Supplementary material for: Epidemiology and Genetic Analysis of SARS-CoV-2 in Myanmar during the Community Outbreaks in 2020
Source: Viruses. 2022 Jan 27;14(2):259. doi: 10.3390/v14020259 (PMC8875553; doi:10.3390/v14020259)
Supplement: Supplementary file 1 [file viruses-14-00259-s001.zip › TableS2.pdf]

Table S2. Demographic and genetic characteristics of GISAID registered COVID-19 sequences from Myanmar from the other study in 2020 [13] [14].

No	Strain name	Age (years)	Sex	Place	Contact history	Travel history (foreign)	Date of RT-PCR test (2020)	Symptoms	Outcome	Next strain clade	GISAID clade	PANGOLIN lineage
1	hCoV-19/ MMC137/ 2020	7	F	Yangon	Yes	No	22-Apr	No	Recovered	19A	O	B.6
2	hCoV-19/ Myanmar/ DMR-MM01 /2020	35	F	Yangon	Yes	No	24-Apr	No	Recovered	19A	O	B.6
3	hCoV-19/Myanmar/DMR-MM02/2020	31	F	Yangon	Yes	No	24-Apr	No	Recovered	19A	O	B.6
4	hCoV-19/ Myanmar/ DMR-MM03 /2020	53	M	Yangon	Yes	No	24-Apr	No	Recovered	19A	O	B.6
5	hCoV-19/ Myanmar/ DMR-MM04 /2020	48	M	Yangon	No	No	3-May	Unknown	Recovered	19A	O	B.6
6	hCoV-19/ Myanmar/ DMR-MM05 /2020	28	F	Yangon	Yes	No	7-May	Sneezing, loss of taste	Recovered	20B	GR	B.1.1
7	hCoV-19/ Myanmar/ DMR-MM06 /2020	41	F	Yangon	No	Yes (India)	25-May	Unknown	Recovered	20A	GH	B.1.36
8	hCoV-19/ Myanmar/DMR-MM07 /2020	29	F	Yangon	No	Yes (China)	2-Jun	Unknown	Recovered	20A	G	B.1.210
9	hCoV-19/ Myanmar/ DMR-MM08 /2020	63	F	Yangon	No	Yes (India)	26-May	Unknow	Recovered	20A	GH	B.1.36
10	hCoV-19/ Myanmar/ DMR-MM09 /2020	66	M	Yangon	No	Yes (India)	26-May	Unknown	Recovered	20A	GH	B.1.36
11	hCoV-19/ Myanmar/ DMR-MM10 /2020	40	F	Yangon	No	Yes (India)	30-May	Unknown	Recovered	20B	GR	B.1.1.174
12	hCoV-19/ Myanmar/ DMR-MM11 /2020	22	M	Sittwe	Yes	No	21-Aug	Unknown	Recovered	20A	GH	B.1.36.16
13	hCoV-19/ Myanmar/ DMR-MM12 /2020	58	M	Sittwe	Yes	No	21-Aug	Unknown	Recovered	20A	GH	B.1.36.16
14	hCoV-19/ Myanmar/ DMR-MM13 /2020	28	M	Sittwe	Yes	No	21-Aug	Unknown	Recovered	20A	GH	B.1.36.16
15	hCoV-19/ Myanmar/ DMR-MM14 /2020	38	F	Sittwe	No	No	20-Aug	Unknown	Recovered	20A	GH	B.1.36.16
16	hCoV-19/ Myanmar/ DMR-MM15 /2020	29	F	Sittwe	No	No	20-Aug	Unknown	Recovered	20A	GH	B.1.36.16

17	hCoV-19/ Myanmar/ DMR-MM16 /2020	83	M	Sittwe	No	No	20-Aug	Unknown	Recovered	20A	GH	B.1.36.16
18	hCoV-19/ Myanmar/ DMR-MM17 /2020	28	F	Sittwe	Yes	No	20-Aug	Unknown	Recovered	20A	GH	B.1.36.16
19	hCoV-19/ Myanmar/ DMR-MM18 /2020	43	M	Sittwe	Yes	No	21-Aug	Unknown	Recovered	20A	GH	B.1.36.16
20	hCoV-19/ Myanmar/ DMR-MM19 /2020	50	F	Sittwe	Yes	No	19-Aug	Unknown	Recovered	20A	GH	B.1.36.16
21	hCoV-19/ Myanmar/ DMR-MM20 /2020	21	F	Sittwe	No	Yes (Philippines)	19-Aug	Unknown	Recovered	20B	GR	B.1.1.263
22	hCoV-19/ Myanmar/ DMR-MM21 /2020	75	M	Yangon	No	No	12-Sep	Unknown	Deceased	20A	GH	B.1.36.16
23	hCoV-19/ Myanmar/ DMR-MM22 /2020	84	F	Yangon	No	No	13-Sep	Unknown	Deceased	20A	GH	B.1.36.16
24	hCoV-19/ Myanmar/ DMR-MM23 /2020	58	M	Yangon	Yes	No	16-Sep	Unknown	Deceased	20A	GH	B.1.36.16
25	hCoV-19/ Myanmar/ DMR-MM24 /2020	67	M	Yangon	Yes	No	16-Sep	Unknown	Deceased	20A	GH	B.1.36.16
26	hCoV-19/ Myanmar/ DMR-MM25 /2020	24	M	Yangon	No	No	14-Sep	Unknown	Deceased	20A	GH	B.1.36.16
27	hCoV-19/ Myanmar/ DMR-MM26 /2020	74	M	Yangon	No	No	17-Sep	Unknown	Deceased	20A	GH	B.1.36.16
28	hCoV-19/ Myanmar/ DMR-MM27 /2020	75	F	Yangon	Yes	No	17-Sep	Unknown	Deceased	20A	GH	B.1.36.16
29	hCoV-19/ Myanmar/ DMR-MM28 /2020	48	F	Yangon	No	No	17-Sep	Unknown	Deceased	20A	GH	B.1.36.16
30	hCoV-19/ Myanmar/ DMR-MM29 /2020	23	F	Yangon	No	No	17-Sep	Unknown	Deceased	20A	GH	B.1.36.16
31	hCoV-19/ Myanmar/ DMR-MM30 /2020	53	M	Yangon	No	No	28-Sep	Unknown	Recovered	20A	GH	B.1.36.16

References

13. Ministry of health and sports Myanmar COVID-19 Surveillance Dashboard. Available online: https://mohs.gov.mm/ (accessed on 14 January 2022).
14. Nyunt, M. H.; Soe, H. O.; Aye, K. T.; Aung, W. W.; Kyaw, Y. Y.; Kyaw, A. K.; Myat, T. W.; Latt, A. Z.; Win, M. M.; Win, A. A.; et al., Surge of severe acute respiratory syndrome coronavirus 2 infections linked to single introduction of a virus strain in Myanmar, 2020. *Sci. Rep.* **2021,** 11, (1), 10203.
