# Supplementary figures and images for: Epidemiology and Genetic Analysis of SARS-CoV-2 in Myanmar during the Community Outbreaks in 2020
Source: Viruses. 2022 Jan 27;14(2):259. doi: 10.3390/v14020259 (PMC8875553; doi:10.3390/v14020259)

**(A)** Number of COVID-19 confirmed cases in Yangon.

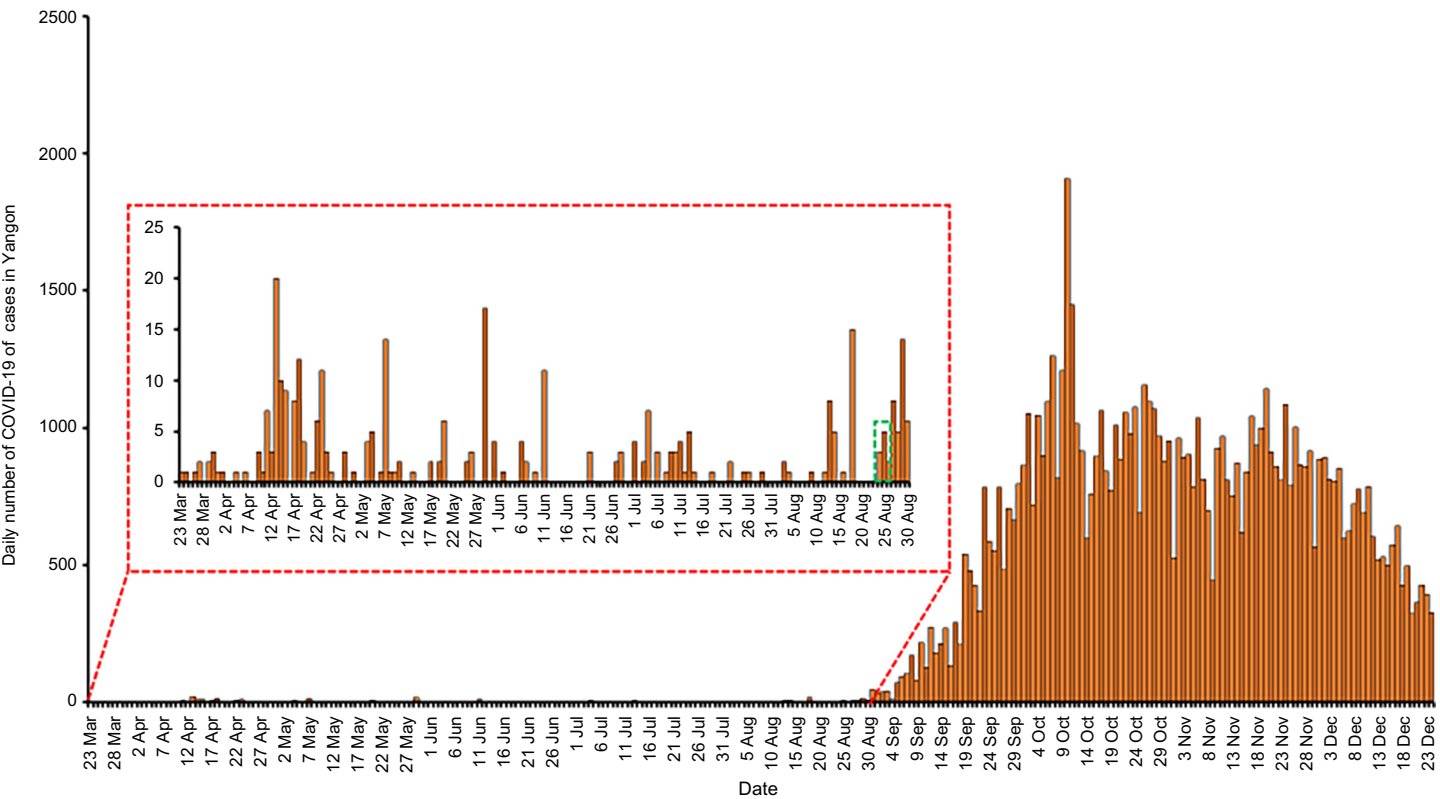

**(B)** Number of COVID-19 death cases in Yangon

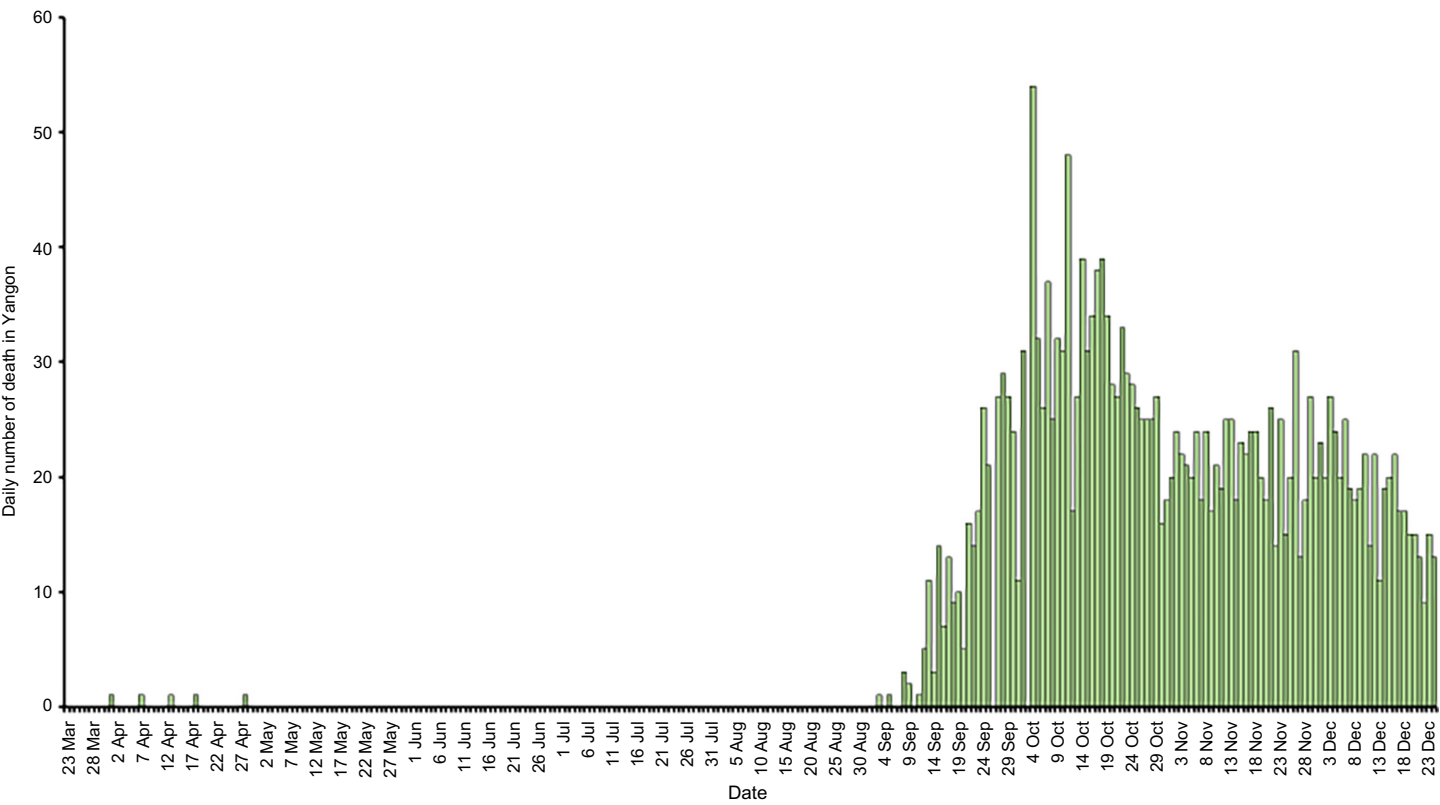

Supplement: Supplementary file 1 [file viruses-14-00259-s001.zip › FigureS1.pdf]
